# Need for addressing oral health disparities in rural Appalachia

**DOI:** 10.15171/hpp.2017.32

**Published:** 2017-09-26

**Authors:** Brian Martin, Amanda H. Wilkerson, Gilbert Patterson, Vinayak K. Nahar, Manoj Sharma

**Affiliations:** ^1^Center for Animal and Human Health in Appalachia, College of Veterinary Medicine, DeBusk College of Osteopathic Medicine, and School of Mathematics and Sciences, Lincoln Memorial University, Harrogate, TN, USA; ^2^Department of Health and Exercise Science, College of Arts and Sciences, The University of Oklahoma, Norman, OK, USA; ^3^Behavioral & Environmental Health, School of Public Health, Jackson State University, MS, USA; ^4^College of Health Sciences, Walden University, Minneapolis, MN, USA

Appalachia is a large region in the eastern United States, spanning 205 000-square-miles from southern New York to northern Mississippi and is home to approximately 25 million people.^1^ It is spread out along the Appalachian Mountains and includes 420 counties in 13 states. While urban centers exist in Appalachia, such as Pittsburgh, the region is characterized by the rural communities in which 42% of the population lives, more than double the national average.^2^ Although the economic disparity of the region has improved in the last century, disparities remain in terms of income, education, access to medical care, and health behaviors.^3^ Rural areas of Appalachia have historically been economically distressed and medically underserved. The poverty level in Appalachian Kentucky is 25.4% and Appalachian Mississippi is 23.4%, which are more than 1.5 times that of the national average.^1^ These disparities also translate into the arena of dental services: there are an average of only 4 dentists per 100 000 individuals in economically distressed regions, compared to the U.S. average of nearly 61 per 100 000.^4,5^

Oral health pertains to the well-being of the oral cavity and pharynx. The most common afflictions are dental caries and periodontal disease, such as gingivitis, while less common but more serious conditions include oral and pharyngeal cancers. Oral health is critical to overall health, as oral disease has been found to be linked to diabetes, heart disease, stroke, and low birth-weight for babies.^6^ Unfortunately, despite the importance of oral health to overall health status, oral health is often overlooked by the public. One study in Appalachia found that residents listed it as their lowest health concern.^7^

Appalachia has higher rates of cancer incidence than non-Appalachian regions, and although the disparity for most forms has improved in recent years, oral cancer rates remain higher than the national average.^2^ Oral cancer rates are also higher among Appalachian vs non-Appalachian regions of the same state.^3^ Oral cancers are particularly dangerous among populations who do not regularly seek screenings and preventative medical care due to its slower onset and initially innocuous symptoms. Symptoms such as a local pain or difficulty chewing are often ignored by individuals living in this region.^4^

Tobacco usage is most often implicated in cases of oral cancer, causing an estimated 75% to 90% of all cases.^4^ It is not surprising, then, that the percentage of current smokers among the Appalachian population is higher than the national average, with approximately 20% of men and 16% of women considered to be smokers,^2^ reaching 26.8% in West Virginia.^3^

Other important issues pertaining to oral health, and certainly more common than oral cancers, are dental caries and periodontal disease. West Virginia leads the nation in edentulism, which afflicts 36% of adults, and reports indicate that only 61% of adults visited a dentist in the past year.^8^ A significant relationship was found between dental fear (e.g. fear of pain or general anxiety towards dental procedures) and delaying dental treatment.^8^ People living in rural Appalachia were also found to have higher rates of oral disease than their urban counterparts. However, among children and adolescents, rates of dental caries did not differ significantly. Rather, rates of unmet dental needs are higher in rural populations, leading to increased rates of oral disease, caries, and edentulism among adults.^9^ Untreated dental pathologies may result in a visit to the emergency department, which not only puts a strain on the healthcare system but also rarely results in definitive treatment.^8^ In the year 2000, the Center for Oral Health Research in Appalachia (COHRA) was established by The University of Pittsburgh and West Virginia University to study the high rates of oral health problems seen in Northern Appalachia.^10^ The first study, COHRA 1, conducted from 2000 to 2010 found high levels of dental caries in children: 5% in 2-year-olds, 21% in 3-year-olds, 35% in 4-year-olds, and 51% in 5-year-olds.^11^ Another study, COHRA 2, is ongoing and involves the assessment of pregnant mothers and their babies.^11^

Another impediment to establishing a pattern of routine treatment is a “present time orientation” (i.e. patients seek treatment the day their symptoms appear, rather than utilizing preventative methods). Present time orientation has been found to be prevalent among Appalachian communities; however, lack of education was found to be more strongly correlated with delayed dental care than any such cultural beliefs.^8^ A recent study found that while 93% accepted that oral health is important, 40% did not brush their teeth at least once daily, and 70% did not floss.^7^ Infrequent tooth brushing has been associated with severe forms of periodontal disease.^12^ Among those who had not seen a dentist in over 2 years, 70% stated that cost or lack of insurance were the reasons.^7^

While recruiting medical professionals to distressed regions can be difficult, there has been an increase in the number of physicians recruited to the Appalachia region in recent years (growth of 11/100 000 compared to the national average of 5/100 000).^4^ Involving primary care physicians in basic dental surveillance, such as checking for caries and giving referrals to dentists, was found to be an effective means of preventing childhood caries, leading some to suggest that oral health competencies be included in the curriculum of non-dental healthcare professionals.^7^

Individual-level health behaviors are largely shared by a community, such that low rates of teeth brushing and high rates of soda consumption are common to many members, illustrating the need for community-wide education initiatives.^13^ Public education initiatives should focus on increasing self-efficacy regarding oral hygiene to increase healthy behaviors such as brushing daily, as well as addressing dental fear so that regular dentist visits may occur. Newer behavioral theories such as the multi-theory model (MTM) of health behavior change^14,15^ should be applied to foster behavior change with regard to tooth brushing and flossing behaviors, particularly in children and adolescents in rural Appalachia, which can lead to inculcation of healthy oral habits. Due to high rates of tobacco usage, efforts should be increased to prevent the initiation of tobacco usage, as well as promoting cessation. Mass media presents a possibility for improving public health behaviors. A 15-week campaign utilizing television and internet advertisements, as well as cooperation from local businesses, was found to successfully decrease daily consumption of sugary beverages (from 57% to 49% of the population) in the Tennessee-Kentucky-Virginia tristate area.^16^ Efforts should be made to increase healthcare access to the underserved communities of Appalachia, with an emphasis on preventative care. As suggested by others, due to the shortage of medical professionals in rural Appalachia, inter-professional cooperation is crucial to helping the individuals of this region.

## Ethical approval

None to be declared.

## Competing interests

Authors declare that they have no competing interests.

## Authors’ contributions

Manuscript conceptualization: VKN and GP; Manuscript writing: BM, AHW, VKN, and MS; Literature review: BM, GP, and MS. Provided suggestions: GP.
